# Mapping the ancestry of primates

**DOI:** 10.7554/eLife.55429

**Published:** 2020-03-03

**Authors:** Ignacio Martínez, Mercedes Conde-Valverde

**Affiliations:** 1 Cátedra de Otoacústica Evolutiva y Paleoantropología, HM Hospitales - Universidad de AlcaláMadridSpain

**Keywords:** hominoids, evolution, phylogenetic tree, inner ear, primates, Other

## Abstract

Structures in the inner ear can help determine the evolutionary relationship between extinct and living primates.

**Related research article** Urciuoli A, Zanolli C, Beaudet A, Dumoncel J, Santos F, Moyà-Solà S, Alba DM. 2020. The evolution of the vestibular apparatus in apes and humans. *eLife* **9**:e51261. doi: 10.7554/eLife.51261

When the term ‘primates’ was originally coined by Carl Linneus back in 1758, it was to classify all species of monkeys, humans and apes into one group based on their anatomical similarities. At that time, the observed similarities were simply a curiosity and did not imply any special relationship between these species. Later, when Charles Darwin published ‘On the Origin of Species’ in 1859, it became clear that species with comparable anatomies are often evolutionarily linked. And when Thomas Huxley published 'Evidence as to Man’s Place in Nature' in 1863, he grouped humans, gibbons, orangutans, gorillas and chimpanzees into a superfamily named the Hominoidea. Since then, understanding the evolutionary relationships within this superfamily has been a fundamental part of research on human evolution.

Extant members of this family, also known as hominoids, can be arranged into two families: the Hylobatidae family, which includes gibbons; and the Hominidae family, which includes orangutans, gorillas, chimpanzees and humans (Figure 1). About 16 to 7 million years ago, during the middle and upper Miocene period, hominoids expanded throughout Europe, Africa and Asia, and diversified into at least 12 different species which have now become extinct (Begun et al., 2012). Fossils from this time led to the identification of a particularly intriguing species called *Oreopithecus bambolii* (Moyà-Solà et al., 2004; Moyà-Solà et al., 2009).

**Figure 1.**
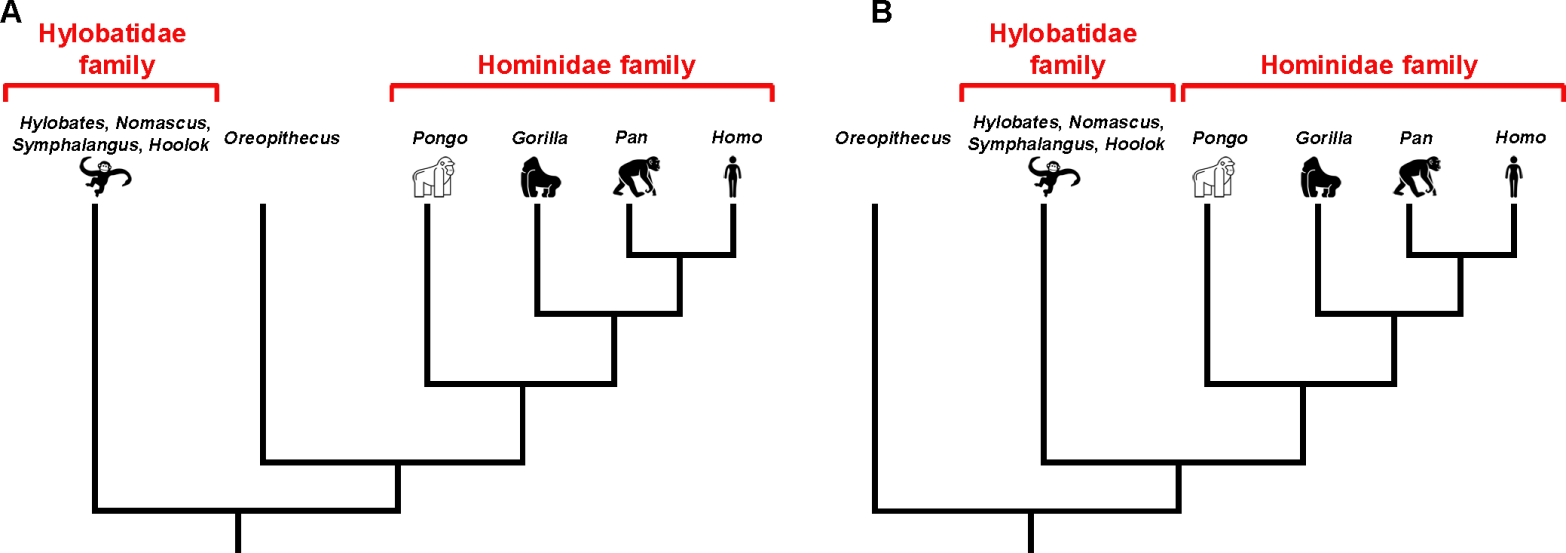
The evolutionary relationship between *O. bambolii* and the Hominidae family. Non-extinct members of the Hominoidea superfamily are split into two families: the Hominidae family, which includes orangutans (*Pongo*), gorillas (*Gorilla*), chimpanzees (*Pan*) and humans (*Homo*); and the Hylobatidae family, which includes various gibbons (*Hylotbates*, *Nomascus*, *Symphalangus* and *Hoolok*). However, it is not clear if the extinct species *Oreopithecus bambolii* split from this branch of the tree of life after the Hylobatidae family split, in which case O. *bambolii* could be part of Hominidae family (**A**), or if it split before the Hylobatidae family split (**B**). Uriciuoli et al. found that O. *bambolii* is not part of the Hominidae family (that is, scenario **B**).

*O. bambolii* fossils date back about 8 million years and come from sites in Sardinia and Tuscany (Rook et al., 2011). The varied features within these fossils have made it difficult to determine the evolutionary history of *O. bambolii* and its relationship to living hominoid species (Harrison and Rook, 1997; Köhler and Moyà-Solà, 1997). As a result, there is an ongoing debate about whether or not *O. bambolii* should be included in the Hominidae family (Begun et al., 2012; Nengo et al., 2017). The key to solving this question is to establish how closely related *O. bambolii* are to the Hominidae family compared to gibbons (Figure 1).

Now, in eLife, David Alba (Institut Català de Paleontologia Miquel Crusafont of the Universitat Autònoma de Barcelona) and colleagues – including Alessandro Urciuoli (Barcelona) as first author, and researchers in France and South Africa – report how studying the shape of semicircular canals in the ears of non-extinct primates can provide a better understanding of how the hominoid family evolved over time (Urciuoli et al., 2020). In recent years, these canals (which form part of the bony exterior of the inner ear) have been used to determine the degree of similarity between members of the Hominidae family (Ponce de León et al., 2018; Quam et al., 2016; Beaudet et al., 2019).

The team reconstructed the three-dimensional shape of semicircular canals of 27 species of living primates and two extinct species, including the *O. bambolii.* This revealed that structures in the inner ear can be used to study the evolutionary relationships between living and extinct hominoid species.

Urciuoli et al. found that although the semicircular canals of *O. bambolii* had similar characteristics to hominoids, this anatomical region had more features in common with two other primate families known as the cercopithecoids and platyrrhines. This suggests that *O. bambolii* are evolutionarily further away from orangutans, gorillas, chimpanzees and humans than gibbons, and therefore cannot be considered a true member of the Hominidae family (Figure 1B).

The next step will be to study the semicircular canals of other extinct hominoid species, and repeat the experiment using other anatomical regions in the inner ear, such as the cochlea.
